# Effectiveness and Safety of Transthoracic Ultrasound in Guiding Percutaneous Needle Biopsy in the Lung and Comparison vs. CT Scan in Assessing Morphology of Subpleural Consolidations

**DOI:** 10.3390/diagnostics11091641

**Published:** 2021-09-07

**Authors:** Marco Sperandeo, Evaristo Maiello, Paolo Graziano, Annalisa Simeone, Salvatore De Cosmo, Lucia Dimitri, Concetta Di Micco, Elio Perrone, Marco Taurchini, Gianmaria Ferretti, Antonio Mirijello, Antonio Varriale, Maria Arcangela Grimaldi, Donato Lacedonia, Carla Maria Irene Quarato

**Affiliations:** 1 Unit of Interventional and Diagnostic Ultrasound of Internal Medicine, IRCCS Fondazione Casa Sollievo della Sofferenza, 71013 San Giovanni Rotondo, FG, Italy; sperandeomar@gmail.com; 2 Unit of Oncology, IRCCS Fondazione Casa Sollievo della Sofferenza, 71013 San Giovanni Rotondo, FG, Italy; e.maiello@operapadrepio.it (E.M.); doctor.dimicco@gmail.com (C.D.M.); 3 Unit of Patology, IRCCS Casa Sollievo della Sofferenza, 71013 San Giovanni Rotondo, FG, Italy; p.graziano@operapadrepio.it (P.G.); lmcdimitri@libero.it (L.D.); 4 Unit of Radiology, IRCCS Casa Sollievo della Sofferenza, 71013 San Giovanni Rotondo, FG, Italy; a.simeone@operapadrepio.it; 5 Department of Internal of Medicine, IRCCS Fondazione Casa Sollievo della Sofferenza, 71013 San Giovanni Rotondo, FG, Italy; s.decosmo@operapadrepio.it (S.D.C.); a.mirijello@operapadrepio.it (A.M.); a.varriale@operapadrepio.it (A.V.); mariella.grimaldi@hotmail.it (M.A.G.); 6 Unit of Nuclear Medicine and PET/TC, IRCCS Fondazione Casa Sollievo della Sofferenza, 71013 San Giovanni Rotondo, FG, Italy; e.perrone@operapadrepio.it; 7 Unit of Thoracic Surgery, IRCCS Casa Sollievo della Sofferenza, 71013 San Giovanni Rotondo, FG, Italy; m.taurchini@operapadrepio.it (M.T.); gm.ferretti@operapadrepio.it (G.F.); 8 Department of Medical and Surgical Sciences, Institute of Respiratory Diseases, University of Foggia, 71122 Foggia, FG, Italy; donato.lacedonia@unifg.it; 9 Institute of Respiratory Diseases, Policlinico “Riuniti” di Foggia, 71122 Foggia, FG, Italy

**Keywords:** transthoracic ultrasound-guided percutaneous needle biopsy, transthoracic ultrasound, chest computed tomography, peripheral pulmonary lesions, diagnostic accuracy, sensitivity, specificity

## Abstract

(1) Background: The aim of this study was to conduct a prospective analysis on the diagnostic accuracy of transthoracic ultrasound-guided percutaneous needle biopsy (TUS-PNB) for the histological assessment of peripheral lung lesions and to assess the performance of transthoracic ultrasound (TUS) examination vs. chest CT (gold standard) in the differentiation between malignant and benign peripheral lung lesions. (2) Methods: A total of 961 consecutive patients with subpleural pulmonary lesions were enrolled. All the patients received a CT scan with contrast; 762 patients underwent TUS-PTNB for suspicion of malignancy, and the remaining 199 enrolled patients underwent only TUS examination as a part of routine follow-up for known non-malignant subpleural consolidations. (3) Results: Among the 762 TUS-guided biopsies, there were 627 (82.28%) malignant lesions, 82 (10.76%) benign lesions, and 53 (6.96%) indeterminate lesions. The overall diagnostic accuracy was 93.04%. The rates of pneumothorax not requiring chest-tube insertion and self-limited hemoptysis were 0.79 and 0.26%, respectively. Patients were divided into two groups based on the benign or malignant nature of the subpleural consolidations. On TUS, both malignant and benign lesions showed mostly irregular margins and a hypoechoic pattern, but no differences were assessed in terms of sonographic margins and pattern between the two groups. There was poor agreement between TUS and chest CT in assessing air bronchograms and necrotic areas. The only finding in the detection of which TUS showed superiority compared to chest-CT was pleural effusion. (4) Conclusions: TUS-PNB was confirmed to be an effective and safe diagnostic method for peripheral pulmonary consolidation, but their sonographic pattern did not allow to rule out a malignant nature. A pre-operative evaluation on CT images, combined with the possibility of performing additional immunohistochemical and cytological investigations and the experience of the medical staff, may improve the diagnostic yield of TUS-guided biopsies.

## 1. Introduction

Pulmonary consolidations are the most common radiological findings encountered in outpatient and inpatient clinical care. If in a patient with a fever and cough, new-onset radiological infiltrates suggest the diagnosis of pneumonia, the investigation of patients with chronic lung lesions may be challenging. Firstly, it must be determined whether the lesion is likely to be benign or malign, secondly it is crucial to determine the histology and the correct stage if lung cancer is confirmed. An initial non-invasive study on the possible nature of the lesion is best done with a CT scan, that allows to explore the whole lung [1,2]. Malignancy characteristics on the chest CT are increased size, spiculated margins, and high growth rate. In addition, malignancy tends to enhance after injection of IV contrast [3]. If lung cancer is the most likely diagnosis and curative resectability is in doubt or lung function testing reveals that the patient is not candidate for surgical resection, the histological diagnosis should be confirmed by the least invasive procedure prior to introduce the appropriate chemotherapy or radiotherapy [1,4,5]. In this context, percutaneous biopsy (PNB) of peripheral lung lesions is a less invasive technique that may play a critical role in obtaining pathologic proof of malignancy, guiding staging, and planning treatment with less complications. In particular, PNB under CT guidance is regarded as the gold standard for peripheral lesions, which cannot be diagnosed with bronchoscopy [6].

Transthoracic ultrasound (TUS) is a safe, radiation-free, rapid, and cost-effective complementary imaging tool that allows the detection peripheral pulmonary nodules or masses when they are adherent to the parietal pleura [7,8]. Currently, TUS guidance has been generally recognized as effective and safe as CT guide for a percutaneous needle biopsy (PNB) of peripheral lung lesions, with the undeniable advantage of real-time visualization of the target lesion. As a result, TUS-PNB has been included in the ERS/ATS [9] and NICE guidelines [10] among the possible methods of choice for histological assessment of subpleural lung lesions. On the other hand, although sonomorphologic criteria to differentiate peripheral lung consolidations have been defined, the effectiveness of TUS examination in the morphological characterization of lung consolidations is certainly less precise than the CT attenuation pattern. Indeed, the TUS examination of subpleural lung lesions is more affected by the difference in the acoustic impedance encountered by the ultrasound beam in crossing the soft tissues of the chest wall that interposes between the probe and the lesion and other limitations inherent the setting of the ultrasound scanner, the positioning of the probe with respect to the patient’s chest and the patient’s degree of collaboration [11,12].

On this background, the primary endpoint of this study was to conduct a prospective analysis on the diagnostic accuracy of TUS-guided PNB for the histological assessment of malignancy in a large series of peripheral lung lesions. The secondary endpoint of the study was to assess the performance of TUS vs. chest CT (gold standard) in the morphological differentiation between malignant and benign peripheral lung lesions.

## 2. Materials and Methods

This was a prospective single-center observational study. From January 2015 to December 2019, we enrolled a total of 961 consecutive patients (mean age: 48.47 ± 14.49 years; M 767, F 194) with subpleural pulmonary lesions scheduled for systematic TUS examination in our Unit of Interventional and Diagnostic Ultrasound of the Research Institute “Fondazione Casa Sollievo della Sofferenza Hospital” (San Giovanni Rotondo, Italy). The inclusion criteria were: (1) age > 18 years; (2) presence of subpleural pulmonary lesions defined as lesions not only abutting the pleura but also having an accessible ultrasound window; (3) availability of a contrast-enhanced computed tomography (CECT). 

A total of 762 patients underwent TUS-PTNB for suspicion of malignancy. Biopsy exclusion criteria included the following: (1) a prolonged prothrombin time (PT-INR > 1.5) or a platelet count < 30,000; (2) right-to-left shunts; (3) severe pulmonary hypertension (i.e., pulmonary artery pressure > 90 mmHg); (4) uncontrolled systemic hypertension (i.e., systolic blood pressure > 140 mmHg); (5) recent myocardial infarction or unstable angina; (6) presence of consciousness and mental disorders; (7) inability to tolerate the operation positions and cooperate with breathing instructions during biopsy. The remaining 199 enrolled patients had known non-malignant subpleural consolidations, including the following: (1) pneumonia, (2) obstructive atelectasis, and (3) compressive atelectasis. Such patients underwent only TUS examination as a part of routine follow-up.

The primary endpoint of our study was to analyze the effectiveness and safety of TUS-guided PNB in the subgroup of patients who needed a histological assessment for suspicion of malignancy. Excluding the patients who received an inconclusive diagnosis from TUS-PNB, the remaining enrolled patients were then divided into groups based on the benign or malignant nature of their subpleural consolidations. The secondary endpoint was to assess the performance of TUS vs. chest CT (gold standard) in the morphological characterization of peripheral lung lesions in the two groups. 

All the participants provided informed written consent for all procedures, including biopsy. The study followed the amended Declaration of Helsinki and the local institutional Ethical Review Board approved the protocol (TACE-CSS, n 106/2018).

### 2.1. Contrast-Enhanced Chest CT (CECT) 

All the patients received a CT scan with contrast within 7-days before the TUS study and/or the TUS-guided biopsy procedure. Patients with malignant or suspicious for malignancy lesions have been yet examined by a contrast-enhanced CT scan, according to the current diagnostic and staging protocol for lung cancer [1,2]. In patients with clinical-radiological evidence suggestive for infectious pneumonia or atelectasis a contrast-enhanced chest CT scan was performed to further delineate the lesion/s after TUS examination.

Chest CT imaging was performed using a multi-detector CT scanner with 64 channels (Toshiba, Tokyo, Japan). The detailed protocol parameters for CT acquisition were as follows: tube voltage, 120 kVp; standard tube current, 60–120 mAs (using an automatic exposure control system); slice thickness, 0.5 mm; reconstruction interval, 0.5–1.0 mm. Patients in the supine position were asked to hold their breath during scanning. All the patients received a dose 0.5–2 mL/kg of the non-ionic iodine contrast agent *Iopamiro* 370 mg/mL (Bracco, Milan, Italy) IV. The enhanced CT scan started 60 s after the administration of the contrast medium.

A “pulmonary consolidation” was defined as a homogeneous increase in pulmonary parenchymal attenuation obscuring the margins of vessels and airway walls [13]. Pulmonary consolidations were defined “subpleural” when the consolidative process reached the parietal pleural surface. The following characteristics were recorded for each lesion: size (defined as the mean between its maximum and minimum diameter on an axial scan) and margins; presence/absence of air bronchogram, defined as a pattern of air-filled (low-attenuation) bronchi on a background of opaque (high-attenuation) airless lung [13]; presence/absence of necrosis, defined as distinct areas of low attenuation on CT scan, and presence/absence of additional pleural effusion. CT scans were reviewed by two expert radiologists to reach a consensus.

### 2.2. Transthoracic Ultrasound (TUS)

TUS examination was performed by an Esaote MyLab-9 scanner (Esaote-Biomedica, Genoa, Italy) using a convex multi-frequency probe (2–8 mHz). The detailed machine settings for US imaging acquisition were as follows: depth varying between 70–140, time gain compensation (TGC) no more than 50%, focus pointed at the hyperechoic pleural line, activation of the tissue harmonic imaging. Patients were examined in a sitting or semi-sitting position. Each lung field was systematically explored, from the base to the apex, along the anatomical demarcation lines of the thorax. More specifically, exploration was conducted with intercostal longitudinal and transversal scans along the para-vertebral, hemi-scapular, and posterior-axillary lines, posteriorly, along the middle-axillary line, laterally, and along anterior-axillary, hemi-clavicular and para-sternal lines, anteriorly.

The following characteristics were recorded for each lesion: size; regular/irregular margins, and ultrasound pattern that was classified as “hypoechoic” or “mixed” (i.e., hyper/hypoechoic). Lesion size was defined as the mean between its maximum and minimum diameter on a longitudinal TUS scan. The sonographic “air bronchogram” was defined as hyperechoic linear or lenticular spots inside a consolidation. “Necrosis” was identified as focal anechoic areas within a consolidation and presence/absence of pleural effusion. The lack of central color Doppler flow within an identified hypo/anecoic area was used to support the diagnosis of necrosis.

Recorded video clips for each subject were reviewed by three expert sonographers, to reach a consensus. All the sonographers were blinded to concurrent CT scan results.

### 2.3. Transthoracic Ultrasound-Guided Percutaneous Needle Biopsy (TUS-PNB)

TUS-guided PNBs were performed by a radiologist and an internist with over 30 years of experience in interventional ultrasound. All biopsies were performed with the same ultrasound scanner used for TUS examination (Esaote MyLab-9, Esaote-Biomedica, Genoa, Italy) using a 3.5–8 MHz dedicated convex-array puncture probe. The probe was equipped with a holed guide for needle insertion of various angle selections (0°, 15°, and 30°). The biopsy technique employed is called “modified Menghini” and consists of the use of a semi-automatic 18 gauge needle with a Menghini type tip and a pyramidal stylet connected to a syringe plunger (Biomol, Hospital Service SpA, Aprilia, Latina, Italy). With the help of a spring mechanism, the syringe plunger can be charged and released automatically. The operators carried out a careful study of the pre-operative CT images for each lesion before proceeding with the guided procedure.

Local anesthesia was obtained by applying a solution of 20 mg/mL lidocaine cloridrate. Once the lesion was clearly individuated on B-mode TUS scan, the patient was asked to hold their breath and the needle, with the syringe plunger charged, was advanced through the obliged path designed by the dedicated probe and, so, within the lesion under real-time guidance. The syringe plunger was released, removing the stylet and applying suction. The needle was then pushed in and out of the lesion, thus facilitating the ascent of pathological tissue. Biopsy specimens and cell-blocks for both histology and cytology assessment were obtained.

**Figure 1:**
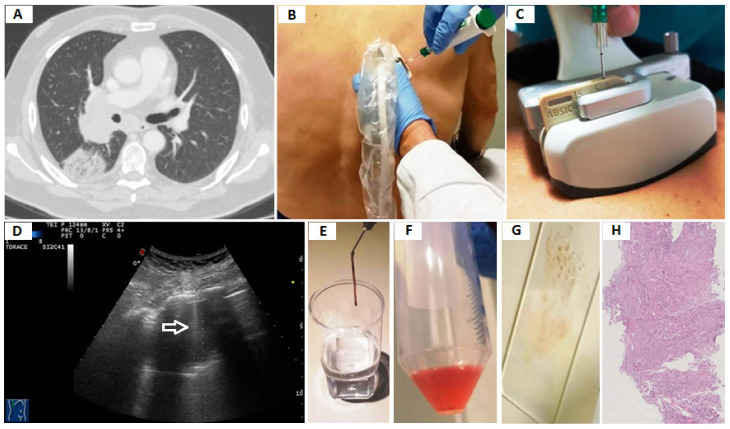
(**A**) Axial chest computed tomography (CT) showing a subpleural pulmonary lesion with inner air bronchograms in the superior segment of the right lower lobe. (**B**) Transthoracic ultrasound-guided percutaneous needle biopsy (TUS-PNB) of the lesion with the patient in a sitting position. (**C**) Dedicated ultrasound convex transducers with a central hole for needle set insertion during TUS-PNB. (**D**) Transthoracic ultrasound scan (TUS) using the dedicated convex probe (3.5–8 MHz) during the US-guided biopsy allowing real-time visualization of the needle (white arrow) in a hypoechoic subpleural lung lesion. (**E**) A specimen suitable for histological assessment. (**F**) A 40–50 mL container filled with a preservative solution for cell-blocks. (**G**) Smear for immediate cytological evaluation. (**H**) The histological examination shows a predominant acinar pattern (hematoxylin and eosin X10). The final diagnosis was pulmonary adenocarcinoma.

A pathologist was not present during the procedure. After each biopsy, the specimen was put on a small piece of sterile gauze and was examined by gross inspection by the operator to judge its quality and determine whether further sampling was required. If the specimen presented a complete, solid strip, the operator supposed that the quality of the specimen was good and that there was a high probability of having punctured an area of viable tissue. If the specimen presented as black, purulent, fragile, fragmented, or liquid, the operator assumed that the specimen was unsatisfactory and there was a high probability of having punctured an area of necrotic or fibrotic tissue. In these cases, the biopsy procedure was repeated in the same single session. The resected specimens were immediately put in 10% formaldehyde solution for histopathology and histochemistry examinations. The remnant tissue in the biopsy needle was partly smeared on a glass slide for immediate cytological evaluation and partly collected into a 40–50 mL container and filled with a preservative solution in order to obtain cell-blocks (Figure 1).

At the end of the biopsy procedure, patients were closely monitored for 3–4 h in order to exclude post-operatory complications. Expiratory chest-X-rays were also performed in order to rule out pneumothorax.

### 2.4. Final Diagnosis

Histo-cytological diagnoses were made by two pathologists with expertise in lung cancer. Speciments were considered to be non diagnostic if the biopsy material was insufficient or it did not allow a clear descriptive diagnosis, such as necrosis, fibrotic tissue, chronic inflammation, or normal lung. Cases with non-diagnostic results were confirmed to be malignant from other means, such as video-assisted thoracoscopic surgery (VATS) or open surgery.

In patients who did not undergo TUS-PNB the benign nature of subpleural consolidations was diagnosed on the basis of the clinical-radiological examination. The diagnosis of pneumonia was confirmed by clinical course or follow-up images that showed the lesions had subsequently disappeared or were significantly reduced in size after starting the appropriate antibiotic therapy.

### 2.5. Statistical Analysis

Descriptive statistics were expressed as mean ± standard deviation (SD) for continuous variables and as absolute numbers (n) and frequencies (%) for nominal data. Diagnostic accuracy, sensitivity, specificity, positive predictive value, negative predictive value, and negative likelihood ratio for TUS-PNB were calculated with a 95% confidence interval (CI). In addition, the empiric Receiver Operating Characteristic (ROC) curve analysis was employed to study the diagnostic performance of TUS-PNB in diagnosing malignant lesions. Lesion size was divided into three groups that measured <2.0 cm, 2–5 cm, and >5.0 cm, according to Chest CT. A one-way ANOVA test was used to compare the rate of indeterminate biopsy in such three groups. A *p*-value of less than 0.05 was considered statistically significant. The overall complication rate of TUS-PNB was evaluated.

Patients who had a definitive diagnosis were divided into two groups based on the benign or malignant nature of the subpleural consolidations. The difference between the two groups and comparison between TUS and chest CT findings were assessed. Statistical analyses were performed using the Student t-test for continuous numerical variables and Fischer exact test for categorical variables. A *p*-value of less than 0.05 was considered statistically significant. Concordant and discordant results between chest CT and TUS in assessing the air bronchogram, necrosis, and pleural effusion were analyzed with a 2 × 2 correlation matrix. The agreement was quantified by the Cohen’s k coefficients, with k values from 0.81 to 1.00 indicating almost perfect agreement; 0.61 to 0.80, substantial agreement; 0.41 to 0.60, moderate agreement; 0.21 to 0.40, fair agreement; 0.01 to 0.20 slight agreement, and less than 0, no agreement. TUS sensitivity, specificity, positive and negative predictive values, and positive and negative likelihood ratios in detecting such findings compared to chest CT were calculated with a 95% confidence interval (CI). The ROC curve analysis was used to study the diagnostic performance of LUS vs. chest-CT. We defined area under the ROC Curve (AUC) values of 0.50–0.59, 0.60–0.69, 0.70–0.79, and ≥0.80 as none, poor, acceptable and excellent discrimination, respectively.

## 3. Results

### 3.1. Diagnostic Yield of TUS-Guided PNB in Diagnosing Peripheral Pulmonary Malignant Lesions

A total of 762 patients (mean age: 47.80 ± 14.42 years; M 622, F 140) underwent TUS-PTNB for suspicion of malignancy. Patient demographic characteristics, lesion characteristics, and biopsy details are summarized in Table 1. In patients with multiple pulmonary lesions at chest CT scan, the one that was most clearly seen at TUS was selected as the main target for biopsy.

**Figure 2:**
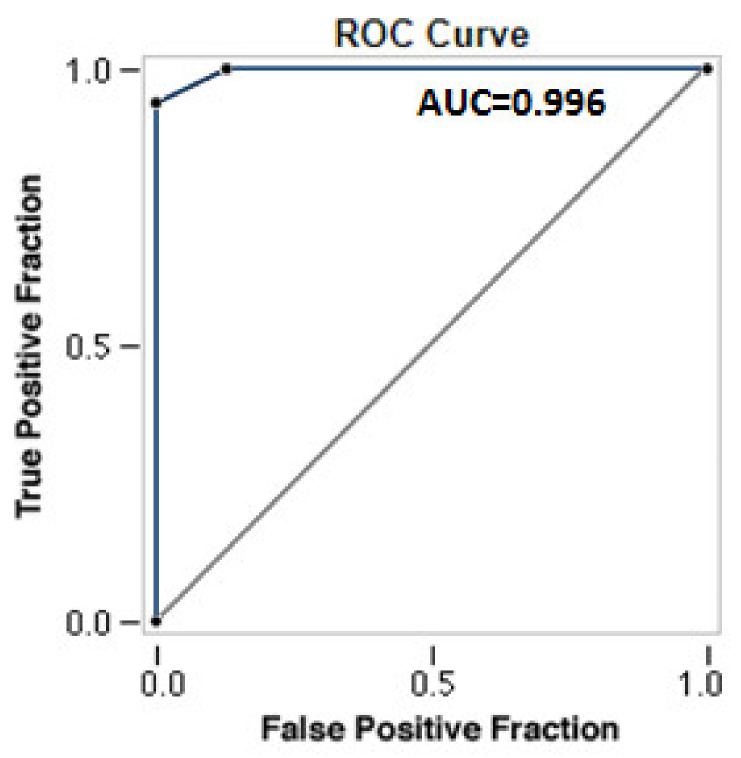
ROC Curve for TUS-PNB in diagnosing the malignant lesions.

We found 627 specific malignant lesions (true-positive cases) and 82 specific benign lesions (true-negative cases) by TUS-guided biopsies. The remaining 53 inconclusive biopsy results were later identified as definitively malignant according to surgical resection (false-negative cases). As a result, the overall diagnostic rate of TUS-PNB in diagnosing the malignant lesions was 93.04% (95% CI: 91.00 to 94.75%), with 92.21% sensitivity (95% CI: 89.93 to 94.11%), 100.00% specificity (95% CI: 95.60 to 100.00%), 100.00% positive predictive value, 60.74% negative predictive value (95% CI: 54.44 to 66.71%) and 0.08 (95% CI: 0.06 to 0.10) negative likelihood ratio. An AUC value of 0.996 (95% CI: 0.993 to 0.999) highlighted an excellent diagnostic yield for TUS-PNB (Figure 2).

The histopathology results from the 762 TUS-guided PNB procedures are presented in Table 2.

Macroscopically inadequate sampling for which the operator deemed necessary the immediate repetition of the biopsy procedure during the same session occurred in 24 (3.15%) cases. The mean number of needle passes per biopsy was 2.75 ± 0.56.

We observed no statistically significant difference in terms of TUS-PNB diagnostic accuracy among different lesion sizes measured on CT scan (*p* = 0.09), despite a mild higher rate of inconclusive biopsies among lesions sized > 5 cm (9/88, 10.23%) followed by lesion sized < 2 cm (11/140, 7.86%). The main causes of inconclusive results were mainly sampling of necrosis for lesions > 5 cm (in 8/11 cases) and sampling of normal (8/9 cases) or inflamed parenchyma (1/9 cases) for lesions sized < 2 cm.

There were no major complications resulting from TUS-PNB. Only 7 (0.79%) cases of partial pneumothorax, not requiring placement of a chest tube, were diagnosed on post-operatory expiratory chest-X-rays. 2 (0.26%) patients presented self-limited hemoptysis with blood-tinged sputum, not requiring medication. No cases of intrapleural hemorrhage occurred.

### 3.2. Diagnostic Accuracy of TUS Examination in Characterizing Peripheral Pulmonary Lesions vs. Chest CT Scan

Excluding the 53 patients who received an inconclusive diagnosis from TUS-PNB, the remaining 908 enrolled patients were divided into groups based on the benign or malignant nature of the subpleural consolidations. In the group of malignant lesions were included 627 patients (mean age: 47.96 ± 14.63 years; M 511, F 116)—of them, 18 were diagnosed with lung metastasis, 49 had small cell lung carcinoma, 89 had squamous carcinoma, 305 had adenocarcinoma, and 116 had large cell undifferentiated carcinoma. In the group of benign lesions were included 281 patients (mean age: 50.28 ± 14.36 years; M 212, F 69). Of them, 164 were diagnosed with pneumonia, 53 had organizing pneumonia, 29 had lung abscess, 21 had obstructive atelectasis, and 14 had compressive atelectasis. Patient demographic characteristics and lesion characteristics on TUS and chest CT scans are summarized in Table 3.

We did not find any statistically significant difference between the mean diameter of malignant and benign lesions, when measured both on chest CT scan or TUS (3.21 ± 0.97 vs. 3.20 ± 0.86; *p* = 0.88 and 3.17 ± 0.99 vs. 3.15 ± 0.89; *p* = 0.77, respectively). Although lesions appeared slightly smaller on TUS, the difference was not statistically significant in both the groups (3.21 ± 0.97 vs. 3.17 ± 0.99; *p* = 0.53 and 3.38 ± 0.86 vs. 3.15 ± 0.89; *p* = 0.50, respectively).

**Figure 3:**
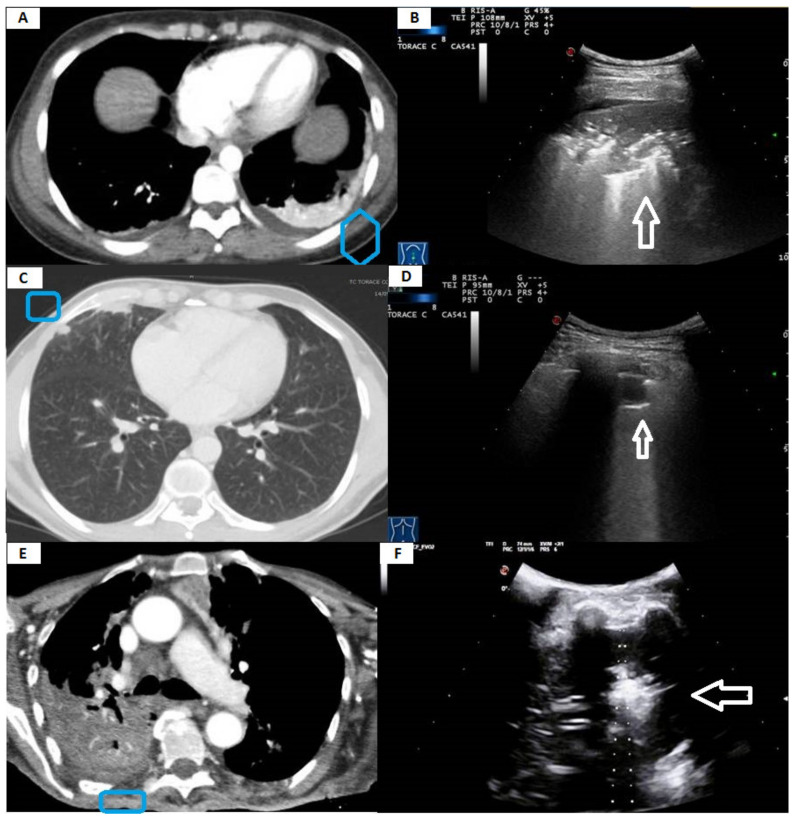
(**A**) Axial chest computed tomography (CT) showing a subpleural pulmonary consolidation (pneumonia) with irregular margins and inner areas of inhomogeneous attenuation (blue box). (**B**) The corresponding transthoracic ultrasound scan (TUS) with a convex probe (3.5–8 MHz) shows a hypo-hyperechoic subpleural lesion with irregular margins (white arrow). (**C**) Axial chest computed tomography (CT) showing a small subpleural pulmonary lesion (histological diagnosis: metastasis from clear cell papillary renal cell carcinoma) with homogeneous attenuation and regular margins (blue box). (**D**) The corresponding transthoracic ultrasound scan (TUS) with a convex probe (3.5–8 MHz) shows a hypo-anechoic subpleural lesion with regular margins (white arrow). (**E**) Axial chest computed tomography (CT) showing a large subpleural pulmonary lesion (histological diagnosis: undifferentiated lung carcinoma) with irregular margins and inner areas of inhomogeneous attenuation (blue box). (**F**) The corresponding transthoracic ultrasound scan (TUS) with a convex probe (3.5–8 MHz) shows a mixed hyper-hypoechoic subpleural lesion with irregular margins (white arrow).

On chest CT, malignant lesions showed a statistically significant tendency to have irregular margins (576, 91.87% vs. 85, 30.25%; *p* < 0.0001). In contrast, the benign lesions showed more regular margins (196, 69.75% vs. 51, 8.13%; *p* < 0.0001). This morphological differentiation was not possible in TUS, where malignant and benign lesions showed mostly irregular margins in equal frequency (429, 84.10% vs. 205, 72.95%; *p* = 0.18). On TUS examination, a hypoechoic pattern was the most frequent presenting feature compared to the completely anechoic and the mixed hypo-hyperechoic pattern for both malignant (378, 60.29% vs. 93, 14.83% vs. 156, 24.88%; *p* < 0.0001) and benign (167, 59.43% vs. 29, 10.32% vs. 85, 30.25%; *p* < 0.0001) lesions. Conversely, we recorded no differences in the number of lesions that appeared hypoechoic (378, 60.29% vs. 167, 59.43%; *p* = 0.10), completely anechoic (93, 14.83% vs. 29, 10.32%; *p* = 0.07) or mixed (156, 24.88% vs. 85, 30.25%; *p* = 0.72) between the two groups (Figure 3).

**Figure 4:**
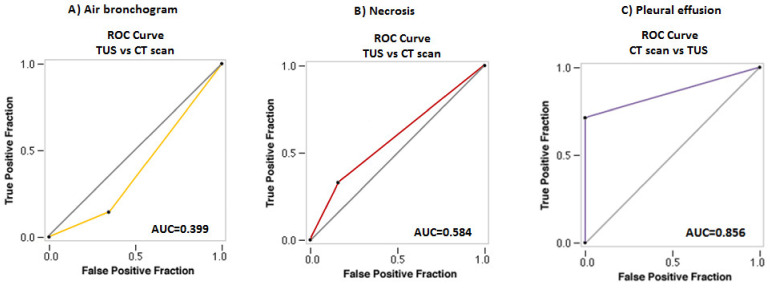
ROC Curve for TUS diagnostic accuracy in diagnosing (**A**) air bronchogram and (**B**) necrosis compared to CT scan. (**C**) ROC Curve for Chest CT diagnostic accuracy in diagnosing pleural effusion compared to TUS.

The presence of air bronchogram was detectable on the chest CT scan in 218 (34.77%) malignant lesions and 82 (29.18%) benign lesions, with no difference between the two groups (*p* = 0.11). Similarly, there was no difference in the rate of inner hyperechoic striae or spot assessed between the two groups of lesions on TUS examination (164, 26.16% vs. 89, 31.67%; *p* = 0.09). The “sonographic air bronchogram” matched with the actual presence of air bronchogram on chest CT scan in 32 malignant lesions and 11 benign lesions (TUS “true positives”). Otherwise, in 132 malignant lesions and 78 benign lesions, TUS examination assessed the presence of hyperechoic striae within the consolidation, but the corresponding chest CT scan was negative for the presence of air bronchogram (TUS “false positives”), and in 186 malignant lesions and 71 benign lesions the chest CT scan assessed the presence of air bronchogram but the corresponding TUS examination was negative (TUS “false negatives”). The Cohen’s k coefficient assessed no agreement between the two diagnostic tests (k= −0.21). TUS showed an overall diagnostic accuracy of 48.57% (95% CI: 45.27 to 51.87%) in detecting air bronchogram, with a sensitivity of 14.33% (95% CI: 10.57 to 18.82%), a specificity of 65.46% (95% CI: 61.53 to 69.24%), a positive predictive value of 17.00% (95% CI: 13.20 to 21.61%), a negative predictive value of 60.76% (95% CI: 58.99 to 62.51%), a positive likelihood ratio of 0.41 (95% CI: 0.31 to 0.56) and a negative likelihood ratio of 1.31 (95% CI: 1.22 to 1.41). An AUC value of 0.399 (95% CI: 0.373 to 0.425) highlighted no discrimination. (Figure 4A).

On chest CT scan, benign lesions showed a higher rate of internal necrosis compared to malignant lesions (104/281, 37.01% vs. 158/627, 25.20%; *p* = 0.0004, respectively). On TUS, the discovery of anechoic regions with no Doppler signal within the consolidation occurred in 132 (21.05%) malignant lesions and 57 (20.28%) benign lesions, with no difference between the two groups (*p* = 0.86). The finding of anechoic areas with no Doppler signal within the consolidation on TUS matched with the actual evidence of necrosis on chest CT in 57 malignant lesions and 29 benign lesions (TUS “true positives”). On the contrary, there were 75 malignant lesions and 28 benign lesions presenting findings of sonographic “necrosis” but in which presence of necrosis was not confirmed by the corresponding CT scan (TUS “false positives”), and 101 malignant lesions and 75 benign lesions where CT scan allowed identification of necrotic areas but the corresponding TUS examination was falsely negative (TUS “false negatives”). According to Cohen’s k coefficient, there was poor agreement between the two diagnostic tests (k = 0.02). TUS showed an overall diagnostic accuracy of 69.27% (95% CI: 66.16 to 72.26%) in assessing necrosis, with a sensitivity of 32.82% (95% CI: 27.17 to 38.87%) and a specificity of 84.06% (95% CI: 81.00 to 86.80%), a positive predictive value of 45.50% (95% CI: 39.46 to 51.68%), a negative predictive value of 75.52% (95% CI: 73.80 to 77.17%), a positive likelihood ratio of 2.06 (95% CI: 1.61 to 2.64) and a negative likelihood ratio of 0.80 (95% CI: 0.73 to 0.88). An AUC value of 0.584 (95% CI: 0.558 to 0.610) highlighted no discrimination (Figure 4B).

On chest CT scan, pleural effusion was detectable in 286/627 (45.61%) malignant lesions and 155/281 (55.16%) benign lesions. TUS examination allowed the detection of pleural effusion in all cases judged positive on chest CT. Furthermore, TUS assessed the presence of mild pleural effusion, not identified on Chest CT scan, in other 131 malignant lesions and 47 benign lesions. According to Cohen’s k coefficient, there was substantial agreement between the two diagnostic tests (k = 0.61). However, TUS showed superiority in the detection of pleural effusion compared to chest-CT. In particular, TUS showed an overall diagnostic accuracy of 100% (95% CI: 99.59 to 100.00%), with a sensitivity of 100% (95% CI: 99.41 to 100.00%) and a specificity of 100% (95% CI: 98.73 to 100.00%); positive and negative predictive values were both 100%. The negative likelihood ratio was 0.00. On the other side, chest CT showed an overall diagnostic accuracy of 80.40% (95% CI: 77.66 to 82.93%) for pleural effusion, with a sensitivity of 71.24% (95% CI: 67.50 to 74.78%), a specificity of 100% (95% CI: 98.73 to 100.00%), a positive predictive value of 100%, a negative predictive value of 61.88% (95% CI: 58.92 to 64.76%) and a negative likelihood ratio of 0.29 (95% CI: 0.25 to 0.33). An AUC value of 0.856 (95% CI: 0.838 to 0.874) highlighted excellent discrimination of chest-CT vs. TUS for pleural effusion (Figure 4C).

Detailed chest CT and TUS findings in malignant and benign lesions included in the study are reported in Table 4 and Table 5.

## 4. Discussion

Our study confirms that TUS-guided PNB is an effective and safe method for sampling TUS-detected pulmonary lesions that are suspicious or highly suggestive of malignancy. On the other hand, our results highlight that TUS is not an accurate imaging method for characterizing peripheral lung lesions compared to CT scans.

Understanding our results requires a preliminary discussion of ultrasound physics. Ultrasound exploration of the lung is limited by two key enemies: bone and air. The high acoustic mismatch between soft tissues of the chest wall and the normally aerated lung causes a subtotal reflection of the ultrasound beam (~96%), preventing the creation of direct imaging of the pulmonary parenchyma. In such conditions, the only detectable findings are a horizontal hyperechoic “pleural line” and other more or less represented reverberation artifacts, classified as horizontal A-lines and vertical B-lines. On the contrary, TUS may be able to visualize peripheral consolidated lung parenchyma when the acoustic impedance posed by air is removed by the replacement with fluid, inflammatory exudates, cellular infiltrates, and/or growing tissue. More specifically, only peripheral consolidations that are strictly adherent to the parietal pleural surface can be imaged because the interposition of even a few mm of air is able to block US signal, thus hiding even very big space-occupying lesions. In addition, the acoustic barrier represented by the bony structures of the thoracic cage reduces the visible pleural surface to 70% [11,12]. As a result, TUS cannot replace chest CT in the examination of the whole lung.

### 4.1. TUS-Guided PNB for the Diagnosis of Peripheral Pulmonary Malignant Lesions

Despite limitations inherent in the method, several studies have reported that TUS guidance for PNB is comparable to chest CT guidance in terms of sample accuracy for pleural or peripheral subpleural lung lesions, showing this procedure a diagnostic yield ranging between 76 and 97.1% [14,15,16,17,18,19,20,21]. Accordingly, the overall diagnostic rate of TUS-guided biopsy in our study was 93.04%.

Lesions >5.0 cm and <2.0 cm were associated with a higher, although not statistically significant, rate of non-diagnostic biopsies compared with lesions sized between 2.0 cm and 5.0 cm. The lower diagnostic accuracy in larger lesions can be justified by the assumption of mistaken sampling caused by the presence of a greater amount of necrosis [22,23,24,25]. Regarding smaller lesions, several reasons have been identified for a higher rate of indeterminate results [26,27]. First, the smaller is the lesion, the more difficult it becomes to accurately center the target and the more likely it becomes to obtain specimens from peripheral areas rather than the lesion itself. Second, even shallow respiratory movements can affect the position of small lesions, thus increasing the possibility of missing the actual target. Third, the smaller the lesion, the greater the possibility to obtain specimens of insufficient volume for histopathology assessment. In the present study, causes of non-diagnostic biopsy were mainly represented by the sampling of normal or inflamed parenchyma for lesions sized < 2 cm (in 29/30 cases) and sampling of necrosis mixed or not with fibrous tissue for lesions > 5 cm (in 14/16 cases), thus confirming these speculations.

Globally, TUS-PNB showed high sensitivity (92.21%; 95% CI: 89.93 to 94.11%) and specificity (100.00%; 95% CI: 95.60 to 100.00%) in allowing histological diagnosis. The obtained ROC curve (AUC: 0.996) confirmed the excellent diagnostic yield of the procedure.

The reasons for our valuable outcome may also be methodological. First, the use of a dedicated probe with a central hole for the introduction of the needle set optimized the procedure, as it allowed to follow the needle in its road in real time (with an image exactly on the line of the target lesion and the transducer) and helped in directing the needle inside the lesion to be biopsied, providing adequate specimens for histological examination and complementary immunohistochemical and immunogenetic tests. Second, each lesion in our study was carefully studied on a pre-operative CT scan before proceeding with the guided procedure. This allowed us to made an a priori evaluation on where to bite the lesion in order to avoid necrosis and to sample viable tissue. Third, the samples were collected by expert operators who, during the execution of the biopsy, were able to notice any lack of hardness within the lesion. In such cases, a repeated “back and forth” movement with the needle could have facilitated the ascent of pathological material along the obliged path of the dedicated probe. Additionally, the experience of the operators enabled them the choice of discarding any sample for which an accurate diagnostic result would not be expected (e.g., in case of macroscopic areas of necrosis or samples that were too small and/or excessively fragmented) and when to immediately repeat the biopsy in the same single session. Fourth, the preparation of the cell-blocks may have improved the diagnostic accuracy, providing specimens suitable also for cytological study. Last but not least, the experience of the pathologists represented another very important factor in ensuring the high diagnostic accuracy of TUS-PNBs in our experience. The safety profile of the study procedure was excellent. There were no major complications, and the occurrence of partial pneumothorax was less than 1%. This could be due to the skills of the operators but also to the use of an atraumatic 18-gauge needle, that allowed obtaining specimens suitable for histologic diagnosis, minimizing at the same time the occurrence of complications, which appear to be more frequent with needles of higher caliber (i.e., 14–16 gauge) [28,29].

### 4.2. TUS Examination in the Morphological Characterization of Peripheral Pulmonary Lesions vs. Chest CT Scan

Some authors assessed that TUS may also help in determining the etiology of pulmonary consolidations with the study of some ultrasound signs, such as the quality of the deep margins [30], the presence of air bronchogram [31], or the vascular pattern within the consolidation [32]. However, the actual role of TUS in characterizing the morphology of lung subpleural lesions is still debated. Therefore, in the present study, we also conducted a systematic analysis on TUS accuracy in the characterization of lung lesions. Chest CT scan was regarded as the standard gold method against which to compare TUS findings.

In general, the size of the consolidations appeared smaller on TUS than on the chest CT, despite the difference was not statistically significant. The main explanation for this result is that the transition area between the consolidation and the healthy parenchyma is more air-filled, which results in ultrasound artifacts reducing the complete view of the periphery of the lesion at TUS. On the chest CT, malignant lesions showed a statistically significant tendency to have irregular margins compared to benign lesions. On the contrary, this morphological differentiation was not possible in TUS, where malignant and benign lesions showed an equal frequency of irregular margins. Once again, the explanation for this result relies upon the fact that the more air-filled periphery of the consolidation creates ultrasound artifacts at the interface between the consolidated lung and the healthy lung parenchyma, resulting in irregular and blurred deep margins. This sign, also called “shred” or “fractal” sign, is not seen in lobar consolidations, where deep borders may be linear and well defined, as consolidated and aerated lung lies adjacent on opposite sides of the pleural fissures.

**Figure 5:**
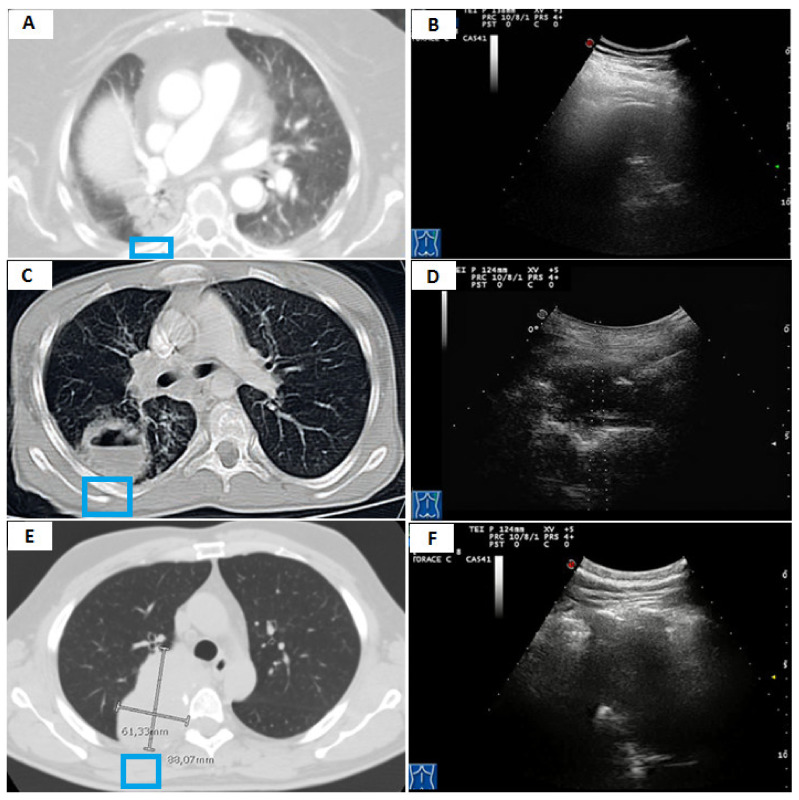
(**A**) Axial chest computed tomography (CT) showing a subpleural pulmonary consolidation (pneumonia) with inner air bronchograms (blue box). (**B**) The corresponding transthoracic ultrasound scan (TUS) with a convex probe (3.5–8 MHz) shows a hypoechoic subpleural lesion presenting a single hyperechoic stria. (**C**) Axial chest computed tomography (CT) showing a pulmonary abscess (blue box). (**D**) The corresponding transthoracic ultrasound scan (TUS) with a convex probe (3.5–8 MHz) shows a hypo-anechoic subpleural lesion with inner hyperechoic spots. (**E**) Axial chest computed tomography (CT) showing a large expansive pulmonary lesion whose histological diagnosis was lung adenocarcinoma (blue box). (**F**) The corresponding transthoracic ultrasound scan (TUS) with a convex probe (3.5–8 MHz) shows a not completely viewable hypoechoic subpleural lesion.

The sonographic pattern did not allow to distinguish between malignant and benign lesions. A hypoechoic appearance was the most frequent sonographic pattern compared to the others in both malignant and benign lesions. According to the available literature, pneumonia may appear on TUS as a mixed hyper/hypoechoic (i.e., “hepatized”) or hypoechoic consolidations of varying size and shape [33,34]. A completely anechoic consolidation may be regarded as indicative of necrotizing pneumonia. In addition, pulmonary abscesses usually appear as anechoic lesions with or without visible hyperechoic echoes inside due to the collections of corpuscular fluid [35]. The sonographic morphology of the atelectatic lung resembles that of other hypoechoic or mixer hypo-hyperechoic consolidations in appearance. Compression atelectasis is easily seen below a pleural effusion, which also acts as an excellent acoustic window. Obstructive atelectasis can be detected only if the collapsed area of the lung parenchyma extends to the pleural line [11,12]. On the other hand, lung tumors are usually hypoechoic, but hyperechoic and anechoic patterns (due to the presence of colliquative necrosis) are not infrequent [36]. The sonographic pattern observed in benign and malignant lesions included in our study was perfectly consistent with what is described above (Figure 5).

**Figure 6:**
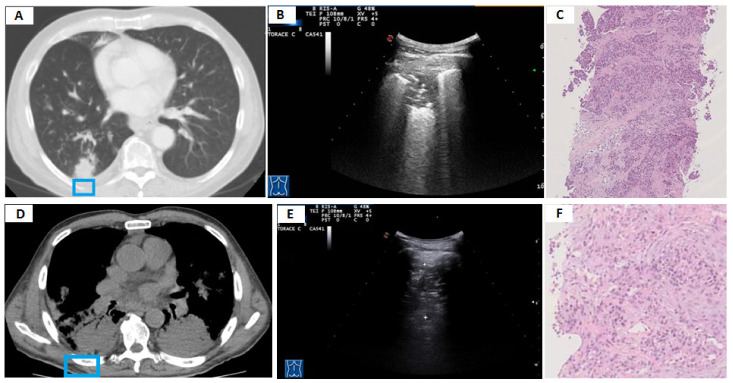
(**A**) Axial chest computed tomography (CT) showing a subpleural pulmonary lesion without inner air bronchograms in the right lower lobe (blue box). (**B**) The corresponding transthoracic ultrasound scan (TUS) with a convex probe (3.5–8 MHz) shows a hypoechoic subpleural lesion presenting inner air “bronchograms-like” hyperechoic spots and striae. (**C**) The histological examination showed a predominant solid pattern without keratinizing or glandular differentiation (hematoxylin and eosin X10). The final diagnosis was non-small-cell lung carcinoma (NSCLC), not other specified (NOS). (**D**) Axial chest computed tomography (CT) showing bilateral subpleural pulmonary consolidations. The consolidation highlighted by the blue box presents inner air bronchograms and some portions with an incomplete contact to the parietal pleura. (**E**) The corresponding transthoracic ultrasound scan (TUS) with a convex probe (3.5–8 MHz) shows a hypoechoic subpleural lesion with inner hyperechoic spots and striae. (**F**) The histological examination shows a mixture of inflammatory cells and fibroblastic plugs within airspaces (hematoxylin and eosin X10). The final diagnosis was organizing pneumonia.

The sonographic “air bronchogram” is described as the presence of hyperechoic spots or stripes within a consolidation, moving or not, with a respiratory excursion. The genesis of this ultrasound finding has been attributed to a change in acoustic impedance between the consolidated lung and air-filled bronchi, and some authors have attributed to such ultrasound findings high specificity and positive predictive value in diagnosing pneumonia [31]. However, the presence of air bronchogram was detectable on chest CT scan in both malignant and benign lesions in our study, with no difference between the two groups. Accordingly, different studies in the literature have shown that even in lung carcinomas, it is possible to see areas of CT air bronchogram, confirming that this finding cannot be considered as a reliable marker of benign consolidation [37,38]. The presence of a “static” air bronchogram on TUS has also been regarded as a useful sign in distinguishing between obstructive atelectasis (caused by lower airway obstruction) and other consolidations (supplied by patent bronchi) [31]. Anyhow, the simple change in angulations of the probe and the patient’s respiratory rate may increase the perceived occurrence of movement, making the discrimination process between the dynamic and static sonographic “air bronchogram” only a subjective and not reproducible overview. Furthermore, no study or meta-analysis so far demonstrated that such lenticular or arborescent hyperechoic images on TUS do really correspond to the CT imaging finding of air bronchogram [39]. As a matter of fact, in our study, Cohen’s k assessed no agreement between the two diagnostic methods. This finding showed a sensitivity of only 14.33% and a positive predictive value of only 17.00% in assessing the actual CT-detected air bronchogram. Indeed, TUS may not be able to highlight this finding when that portion of the bronchial tree is three-dimensionally localized in a plane that is deeper than the two-dimensional plane at the level of which the ultrasound beam cuts the lesion. In addition, ultrasound may not identify portions of the lesion that are hidden by the bony structures of the rib cage. On the other hand, if it is true that hyperechoic spots or striae inside a consolidation may match with the actual presence of an air bronchogram on Chest CT, it is also true that TUS may result falsely positive for the presence of hyperechoic spots or striae also due to an incomplete contact of the consolidation with the parietal pleura allowing the interposition of few microns of air between different areas of the studied lesion [22]. Moreover, some hyperechoic spots and striae may be actually related to the heterogeneous inner structures of lung neoplasm and chronic benign lesions, such as organizing pneumonia, where we may find the alternation between areas of the consolidated lung with micro-areas of colliquative necrosis and/or plugs of fibrous tissue [40]. These assumptions may explain the specificity of only 65.46% for the TUS finding of “sonographic air bronchogram” vs. CT scan (Figure 6).

**Figure 7:**
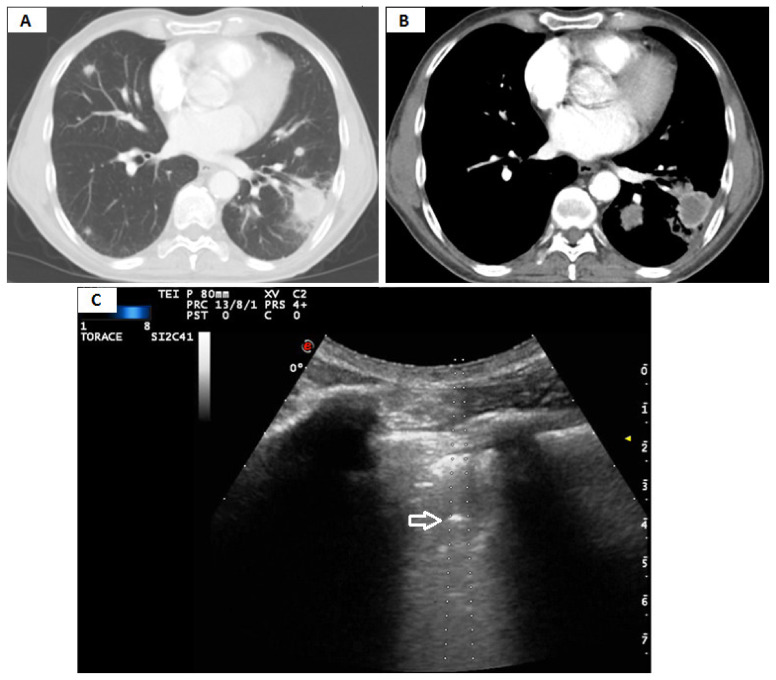
(**A**) Axial chest computed tomography (CT) (pulmonary window) showing a subpleural pulmonary lesion in the left lower lobe whose final histological diagnosis was a metastasis from renal carcinoma. (**B**) The mediastinal window of the same CT scan shows internal colliquation of the lesion. (**C**) The corresponding transthoracic ultrasound scan (TUS) with a convex probe (3.5–8 MHz) during the US-guided biopsy allows the real-time visualization of the needle tip (white arrow) in a mixed hypo-hyperechoic subpleural lung lesion.

The discovery of anechoic regions with no Doppler signal within the consolidation showed a low sensitivity (32.82%) in assessing necrotic areas, with only a slight agreement between TUS and Chest CT. Once again, false negatives can be justified by the position occupied by the areas of necrosis within the consolidation and by the physical limitations encountered by ultrasound in the study of lung lesions [22] (Figure 7).

In addition, false negatives for necrosis may be related to the presence of “flash artifacts” (i.e., a burst of color signal caused by the motion of the transducer or the patients’ breathing) in most patients [41].

The only finding in the detection of which TUS showed superiority compared to chest-CT was pleural effusion. The explanation for such result may rely on the accumulation by gravity near the costo-diaphragmatic sinus or the near the pleural fissures of small effusions that are therefore detectable only during TUS examination in a sitting position, being instead layered in the supine position during CT scan.

The strength of our study is to have used a prospective design in conducting an analysis on the efficacy and safety of TUS-PNB for the histological assessment of subpleural consolidations and on the accuracy of TUS examination in characterizing the morphology of these lesions compared to chest CT in a large number of cases. Indeed, almost all the studies on TUS-PNB in the current literature have a retrospective design that leaves the possibility of residual confounding. The main limitation of our study is that the ideal sample size for both benign and malignant lesions was not pre-established. As a result, the number of benign lesions was far less than that of malignant lesions. This limitation, however, reflects the implementation of TUS examination in an everyday clinical practice, where this imaging method is mostly employed for the biopsy of subpleural lesions suspected for malignancy rather than for the follow-up of benign subpleural lesions. We believe that the findings of our study may offer useful information on the sonographic appearance of lung subpleural lesions and on TUS potentiality in guiding their percutaneous biopsy for histological assessment.

## 5. Conclusions

TUS is an imaging method that has numerous limitations related to the presence of air in the lung and to the obstacle constituted by the bone structures of the rib cage. In addition, TUS findings do not allow to uniquely characterize lesions. The results of our study highlighted that TUS could not exclude the malignant nature of a pulmonary consolidation based on the sonographic size, margins, and pattern alone. In this context, a CT scan represents certainly the gold standard, as it can delineate the distribution and extent of disease, provide clues to narrow the differential diagnosis, and aid in guidance for further invasive diagnostic procedures. Furthermore, ultrasound is a highly operator-dependent method that requires both the appropriate knowledge of ultrasound physics for a correct interpretation and the execution by expert operators. Indeed, although also chest CT is operator dependent in image interpretation, TUS further implies the possibility of high inter- and intra-operator variability in the execution of the examination itself. This variability is linked to several factors, including differences in the setting of the ultrasound scanner, in the positioning of the probe, in the conformation of the patient’s thoracic cage, in the collaboration of the patient (i.e., if the patient has dyspnoea at rest, small lesions can easily escape from the detection), in the presence or not of even a minimal amount of air (also micron) between the pleural surface and the lesion and in the presence of comorbidities that can alter the visualization of the lesion (i.e., fibrosis, emphysema, bronchiectasis, etc.). Anyhow, when subpleural consolidations can be detected on TUS examination, this imaging method can be used to safely and effectively guide a percutaneous needle biopsy for the histological assessment. A pre-operative evaluation of CT images can be of great help in improving the accuracy of TUS-PNB. The experience of operators and pathologists, combined with the possibility of performing additional immunohistochemical and cytological investigations, certainly represent the optimal conditions to guarantee the best diagnostic yield of biopsies.

## Figures and Tables

**Table 1:** Patient demographic characteristics, lesion characteristics, and biopsy details.

Characteristics	N = 762	%
Age	47.80 ± 14.42	
Sex		
Male	622	81.63
Female	140	18.37
Smokers	518	67.98
Patients with multiple lesions on CT scan	590	77.43
Lesion size on CT scan	3.28 ± 1.01	
<2 cm	104	13.65
2 cm–5 cm	570	74.80
>5 cm	88	11.55
Lesion location		
Right upper lobe	80	10.50
Right middle lobe	148	19.42
Right lower lobe	213	27.95
Left upper lobe	103	13.52
Left lower lobe	218	28.61
Repeated biopsy in the single session	24	3.15
Needle passes per biopsy	2.75 ± 0.56	
Diagnostic biopsy	709	93.04
Inconclusive biopsy	53	6.96
<2 cm	11	
2 cm–5 cm	33	
>5 cm	9	

**Table 2:** Histopathology results from TUS-guided PNB.

Final Diagnosis	N = 762	%
Malignant		
Metastasis	18	2.36
Small cell lung carcinoma	49	6.43
Squamous carcinoma	89	11.68
Adenocarcinoma	305	40.03
Undifferentiated carcinoma	166	21.78
Total	627	82.28
Benign		
Organizing pneumonia	53	6.95
Lung abscess	29	3.81
Total	82	10.76
Inconclusive		
Necrotic tissue	8	1.05
Normal lung	10	1.31
Fibrous tissue	14	1.84
Chronic inflammation	21	3.15
Total	53	1.97

**Table 3:** Patient demographic characteristics and lesion characteristics on TUS and chest CT scan in the two groups of malignant and benign lesions.

Variable	Malignant (n = 627)	Benign (n = 281)	*p*-Value
Age, years, (mean ± SD)	47.96 ± 14.63	50.28 ± 14.36	0.03
*Gender:*			
Male n (%)	511 (81.50)	212 (75.44)	0.04
Female n (%)	116 (18.50)	69 (24.56)	
Smokers, n (%)	438 (69.86)	187 (66.55)	0.49
Patients with multiple lesions at CT, n (%)	496 (79.11)	198 (70.46)	0.005
*CT findings*:			
Diameter, cm (mean ± SD)	3.21 ± 0.97	3.20 ± 0.86	0.88
Irregular margins, n (%)	576 (91.87)	85 (30.25)	<0.0001
Smooth margins, n (%)	51 (8.13)	196 (69.75)	
Air bronchogram, n (%)	218 (34.77)	82 (29.18)	0.11
Necrosis, n (%)	158 (25.20)	104 (37.01)	0.0004
Pleural effusion, n (%)	286 (45.61)	155 (55.16)	0.19
*US findings:*			
Diameter, cm (mean ± SD)	3.17 ± 0.99	3.15 ± 0.89	0.77
Irregular margins, n (%)	429 (84.10)	205 (72.95)	0.18
Smooth margins, n (%)	198 (31.58)	76 (27.05)	
Hypoechoic pattern, n (%)	378 (60.29)	167 (59.43)	0.10
Anechoic pattern, n (%)	93 (14.83)	29 (10.32)	0.07
Mixed (hyper/hypoechoic) pattern, n (%)	156 (24.88)	85 (30.25)	0.72
Sonographic “air bronchogram”, n (%)	164 (26.16)	89 (31.67)	0.09
Sonographic “necrosis”, n (%)	132 (21.05)	57 (20.28)	0.86
Pleural effusion, n (%)	417 (66.51)	202 (71.89)	<0.0001

**Table 4:** Detailed chest CT and TUS findings in malignant lesions.

Malignant	Metastasis	Small Cell Lung Carcinoma	Squamous Carcinoma	Adeno-Carcinoma	Undifferentiated Carcinoma
*CT findings*	n = 18	n = 49	n = 89	n = 305	n = 166
Diameter, cm (mean ± SD)	3.33 ± 0.90	3.21 ± 0.89	3.25 ± 0.99	3.24 ± 1.01	3.12 ± 0.91
Irregular margins, n (%)	6 (33.33)	45 (91.84)	86 (96.63)	281 (92.13)	158 (95.18)
Air bronchogram, n (%)	5 (27.78)	8 (16.33)	12 (13.48)	156 (51.15)	37 (22.29)
Necrosis, n (%)	4 (22.22)	27 (55.10)	14 (15.73)	50 (16.39)	56 (33.73)
Pleural effusion, n (%)	4 (22.22)	11 (22.45)	26 (29.21)	146 (47.87)	83 (50.00)
*US findings*					
Diameter, cm (mean ± SD)	3.26 ± 0.93	3.15 ± 0.92	3.20 ± 1.01	3.22 ± 1.03	3.07 ± 0.94
Irregular margins, n (%)	8 (44.44)	43 (87.76)	40 (44.94)	243 (79.67)	95 (57.23)
Hypoechoic pattern, n (%)	9 (50.00)	34 (69.39)	51 (57.30)	181 (59.34)	103 (62.05)
Anechoic pattern, n (%)	3 (16.67)	11 (22.45)	12 (13.48)	12 (3.93)	55 (33.13)
Mixed pattern, n (%)	6 (33.33)	4 (8.16)	26 (29.21)	112 (36.72)	8 (4.82)
Sonographic “air bronchogram”, n (%)	7 (38.89)	9 (18.37)	14 (15.73)	98 (32.13)	36 (21.69)
Sonographic “necrosis”, n (%)	2 (11.11)	32 (65.31)	21 (23.60)	40 (13.11)	37 (22.29)
Pleural effusion, n (%)	6 (33.33)	17 (34.69)	39 (43.82)	217 (71.15)	109 (65.66)

**Table 5:** Detailed Chest CT and TUS findings in benign lesions.

Benign	Pneumonia	Organizing Pneumonia	Lung Abscess	Obstructive Atelectasis	Compressive Atelectasis
*CT findings*	n = 164	n = 53	n = 29	n = 21	n = 14
Diameter, cm (mean ± SD)	3.01 ± 0.75	3.36 ± 0.78	4.07 ± 1.05	3.23 ± 1.02	2.98 ± 0.73
Irregular margins, n (%)	32 (19.51)	47 (88.68)	6 (20.69)	0 (0.00)	0 (0.00)
Air bronchogram, n (%)	67 (40.85)	6 (11.32)	3 (10.34)	4 (19.05)	2 (14.29)
Necrosis, n (%)	52 (31.71)	17 (32.08)	29 (100.00)	4 (19.05)	2 (14.29)
Pleural effusion, n (%)	92 (56.10)	25 (47.17)	16 (55.17)	8 (38.10)	14 (100.00)
*US findings*					
Diameter, cm (mean ± SD)	2.99 ± 0.78	3.26 ± 0.80	4.03 ± 1.10	3.15 ± 1.10	2.91 ± 0.73
Irregular margins, n (%)	128 (78.05)	42 (79.25)	14 (48.28)	21 (100.00)	0 (0.00)
Hypoechoic pattern, n (%)	126 (76.83)	21 (39.62)	0 (0.00)	12 (57.14)	8 (57.14)
Anechoic pattern, n (%)	0 (0.00)	0 (0.00)	29 (100.00)	0 (0.00)	0 (0.00)
Mixed pattern, n (%)	38 (23.17)	32 (60.38)	0 (0.00)	9 (42.86)	6 (42.86)
Sonographic “air bronchogram”, n (%)	36 (21.95)	38 (71.70)	12 (41.38)	1 (4.76)	2 (14.29)
Sonographic “necrosis”, n (%)	34 (20.73)	14 (26.42)	9 (31.03)	0 (0.00)	0 (0.00)
Pleural effusion, n (%)	120 (73.17)	37 (69.81)	20 (68.97)	11 (52.38)	14 (100.00)

## Data Availability

The data presented in this study are available on request from the corresponding author. The data are not publicly available due to ethical reasons.
